# A pharmacist based intervention to improve the care of patients with CKD: a pragmatic, randomized, controlled trial

**DOI:** 10.1186/s12882-015-0052-2

**Published:** 2015-04-16

**Authors:** Danielle Cooney, Helen Moon, Yang Liu, Richard Tyler Miller, Adam Perzynski, Brook Watts, Paul E Drawz

**Affiliations:** Louis Stokes Cleveland VAMC, Cleveland, OH USA; Case Western Reserve University, Cleveland, OH USA; UT Southwestern, Dallas, TX USA; MetroHealth Medical Center, Cleveland, OH USA; Division of Renal Diseases & Hypertension, University of Minnesota, 717 Delaware Street SE, Office 353E, Minneapolis, MN 55414 USA

**Keywords:** Care management, Chronic disease, Hypertension, Randomized trials, Medical informatics

## Abstract

**Background:**

Primary care providers do not routinely follow guidelines for the care of patients with chronic kidney disease (CKD). Multidisciplinary efforts may improve care for patients with chronic disease. Pharmacist based interventions have effectively improved management of hypertension. We performed a pragmatic, randomized, controlled trial to evaluate the effect of a pharmacist based quality improvement program on 1) outcomes for patients with CKD and 2) adherence to CKD guidelines in the primary care setting.

**Methods:**

Patients with moderate to severe CKD receiving primary care services at one of thirteen community-based Veterans Affairs outpatient clinics were randomized to a multifactorial intervention that included a phone-based pharmacist intervention, pharmacist-physician collaboration, patient education, and a CKD registry (n = 1070) or usual care (n = 1129). The primary process outcome was measurement of parathyroid hormone (PTH) during the one year study period. The primary clinical outcome was blood pressure (BP) control in subjects with poorly controlled hypertension at baseline.

**Results:**

Among those with poorly controlled baseline BP, there was no difference in the last recorded BP or the percent at goal BP during the study period (42.0% vs. 41.2% in the control arm). Subjects in the intervention arm were more likely to have a PTH measured during the study period (46.9% vs. 16.1% in the control arm, *P* <0.001) and were on more classes of antihypertensive medications at the end of the study (*P* = 0.02).

**Conclusions:**

A one-time pharmacist based intervention proved feasible in patients with CKD. While the intervention did not improve BP control, it did improve guideline adherence and increased the number of antihypertensive medications prescribed to subjects with poorly controlled BP. These findings can inform the design of quality improvement programs and future studies which are needed to improve care of patients with CKD.

**Trial registration:**

ClinicalTrials.gov: NCT01290614.

**Electronic supplementary material:**

The online version of this article (doi:10.1186/s12882-015-0052-2) contains supplementary material, which is available to authorized users.

## Background

Chronic kidney disease (CKD) is associated with multiple comorbidities including metabolic complications, poorly controlled hypertension, and increased cardiovascular morbidity and mortality [1]. Given the large number of patients with CKD, primary care providers deliver the majority of CKD care [2]. A number of guidelines recommend approaches to the diagnosis, evaluation, and management of patients with CKD [3-6]. Unfortunately, the care of patients with CKD is frequently not adherent to these recommendations; a minority of patients is appropriately monitored for metabolic complications [7]. More importantly, less than half of patients with CKD have well controlled blood pressure (BP) and only about 30% of patients initiate dialysis with a fistula or graft [8-10]. CKD may be the model chronic disease for electronic medical record (EMR) and disease registry based quality improvement because the definition of CKD and many of its accompanying comorbidities and complications are objective and laboratory based.

EMR based approaches to improving the care of patients with chronic diseases have had mixed results. Automated reminders increased rates of compliance with multiple standards of care but improvements waned over time as providers developed “alert fatigue” [11,12]. Adherence to diabetes guidelines improved for medicine residents exposed to performance reports, feedback, and a diabetes registry but no improvement was seen in clinical outcomes [13]. Implementation of an EMR was shown to be associated with increased monitoring of A1c and LDL as well as improvement in A1c levels [14]. With regards to CKD, automated EMR alerts to primary care physicians did not increase renal referrals, assessment of proteinuria, or BP control [15]. We previously demonstrated that giving primary care providers access to a CKD registry does not improve guideline adherence [16]. Successful quality improvement programs likely require strategies designed to overcome multiple barriers as outlined in the Chronic Care Model [17].

As seen nationally, we observed low adherence to CKD guidelines at our institution; therefore, we designed a multifaceted intervention utilizing an EMR based CKD registry to improve the care of patients with CKD. The objective of this pragmatic randomized controlled trial was to evaluate the effect of the quality improvement program on 1) outcomes for patients with CKD and 2) adherence to CKD guidelines in the primary care setting.

## Methods

### Setting and participants

Eligible subjects received primary care from one of 13 Community Based Outpatient Clinics (CBOCs) within the Louis Stokes Cleveland Department of Veterans Affairs Medical Center (VAMC). We included patients if they had 1) moderate to severe CKD defined by a most recent estimated glomerular filtration rate (eGFR), calculated using the 4-variable MDRD equation [18], less than 45 mL/min/1.73 m^2^, 2) a GFR less than 60 mL/min/1.73 m^2^ between 90 days and 2 years prior to the index GFR to ensure the presence of *chronic* kidney disease, and 3) at least one primary care visit in the year prior to study initiation [19]. We excluded patients who had end-stage renal disease (ESRD), were ever referred for hospice care, or were older than 85 years or younger than 18 years. The study was approved by the Cleveland VAMC Institutional Review Board, which granted a waiver of consent. The study adhered to the Declaration of Helsinki.

### Randomization and interventions

In order to evaluate secular trends, two of the 13 CBOCs were randomized to a separate control group using blocked randomization. Patients in these two clinics were not eligible for randomization to usual care vs. the intervention. At study initiation, P.E.D. assigned subjects at the other 11 CBOCs to the intervention and control arms using simple randomization with a blinded computer generated randomization list and a 1:1 ratio.

Participants assigned to the control arm received usual care from their primary care providers. All primary care providers participated in a 45 minute lecture organized around the KDOQI guidelines given at study initiation [4]. The intervention included delivery system redesign which involved engaging pharmacists to interact with patients and collaborate electronically with primary care physicians; self-management support for patients in the form of an informational pamphlet regarding CKD; and a CKD registry.

The CKD registry was established in 2009 and includes all patients with CKD within the Louis Stokes VAMC. Data for the registry was updated nightly from the electronic health record and included demographics, laboratory results, medications, transplant and dialysis status, and information about past and future clinic appointments. The registry was used 1) to identify patients with CKD not receiving guideline adherent care (blood pressure greater than 130/80 mmHg or no parathyroid hormone measured in the past 12 months), 2) by the pharmacists for decision support during the phone call with participants, and 3) to facilitate documentation of the intervention [5,6,17]. Decision support was embedded within the registry in the form of a phone script with branching logic based on patients’ answers (see online Additional file 1). At the completion of the intervention phone call, the registry automatically generated a templated note that was copied into the EMR in the form of a progress note. The registry is not used in daily practice; only study personnel had access to the CKD registry. The study was conducted from February 1, 2011 until January 31, 2012.

The registry was used to identify participants assigned to the intervention arm who had an upcoming primary care appointment. Subjects were sent a letter that described the study and provided a phone number to call if they wished to opt out of the intervention. In the first few weeks of the study, the capacity of the pharmacists to intervene was exceeded and priority for the intervention group was given to participants whose BP was >130/80 mmHg and those who had not been monitored appropriately for metabolic complications.

Prior to study initiation, a standard approach to CKD management based on KDOQI recommendations was developed and reviewed with study pharmacists (D.C. and H.M.). Clinical pharmacists’ scope of practice in nephrology at the VA includes ordering and reviewing labs and prescribing medications. Two study pharmacists, each working approximately one day per week, contacted subjects by phone prior to a primary care appointment to discuss CKD and hypertension. The pharmacists reviewed medications and lifestyle modifications with the patients, ordered KDOQI recommended labs, and arranged nephrology consults for patients with severe CKD (eGFR <30 mL/min/1.73 m^2^). The note included results of the intervention. Once lab results were completed, the pharmacists called patients again to review any abnormal results and initiated appropriate medication changes as needed to treat acidosis, hyperphosphatemia, hyperparathyroidism, Vitamin D deficiency, hyperkalemia, and anemia (see online Additional file 2). Blood pressure medications were not adjusted by the pharmacists but recommendations to primary care providers regarding hypertension management were included in the progress note. A CKD informational packet from the National Kidney Disease Education Program (Chronic Kidney Disease – What Does it Mean for Me?) was sent to each subject in the intervention arm unless they asked that it not be sent (see online Additional file 3).

### Outcomes

The primary clinical outcome was the last clinic systolic BP for patients with poorly controlled hypertension at baseline (>130/80 mmHg). The primary process outcome for all patients was measurement of PTH during the study period. Measurement of PTH was selected because it is specific to the care of patients with CKD and therefore a good measure of CKD guideline adherence. Secondary clinical outcomes were the percentage of patients at goal BP, quality of life, burden of CKD, and incidence of ESRD and all-cause mortality. Secondary process of care outcomes included measurement of phosphorus and urine albumin/creatinine ratio; the number of antihypertensive medications prescribed to those with poorly controlled hypertension; appropriate treatment with ACEI/ARB, phosphorus binders, Vitamin D, and sodium bicarbonate; medication adherence; and the percent of subjects seen by nephrology.

Data were collected from the CKD registry. Personnel responsible for data collection and analysis were blinded to study group assignment. There were no study related clinic visits for this pragmatic trial. Baseline data were defined as the most recent clinic BP or laboratory value within the prior 12 months. Final clinic BP and laboratory values were defined as the last value during the study period. ESRD and all-cause mortality were adjudicated by review of the medical record. In addition to data collected from the CKD registry, 194 subjects (95 from the control group and 99 from the intervention group) were surveyed during the first 3 months of the study (pre-intervention for the intervention group) and again after the study (February-April 2012; 70 control and 76 intervention subjects). Subjects from the intervention and control arms with a primary care appointment during the first 3 months of the study were selected for this additional survey. The phone surveys assessed health related quality of life (SF-12), medication adherence using the Morisky medication scale, Kidney Disease Quality of Life (KDQOL) Short form, health literacy, and the acceptability of the intervention [20-22]. The acceptability and satisfaction with the intervention was assessed after the study using questions rated on a Likert scale (e.g., strongly agree to strongly disagree) as well as opened ended questions (What did you like best/least about being called by the pharmacist?). Of the subjects who were contacted, 194 out of 261 agreed to complete the initial survey.

### Statistical analysis

Primary analyses were based on intention-to-treat and included all randomized participants. Baseline characteristics are reported as mean (standard deviation) and as percentages. For systolic BP, a *t* test was used to evaluate whether the intervention reduced systolic BP. For categorical outcomes (e.g., PTH adherence), the difference between control and intervention arms was compared using a chi-square test. Mixed model regression was utilized for sensitivity analyses to adjust for baseline eGFR and clustering of patients by CBOC and to compare patients randomized to secular control CBOCs to the usual care group in order to assess secular trends. Descriptive data from the patient surveys were analyzed. The impact of the intervention on medication adherence was evaluated using a *t* test. Similarly, the impact of the intervention on quality of life and burden of kidney disease was evaluated using a *t* test. Statistical analyses were performed using R version 2.15 (www.R-project.org) with a 2-sided significance threshold of *P* < .05.

The study was designed to have 80% power to detect a 3.0 mmHg difference in systolic BP between intervention and control groups based on an alpha of 0.05, standard deviation of 15 mmHg, and an estimate of 400 patients per group with poorly controlled BP. For the primary process outcome, assuming 15% of subjects in the control arm would have a PTH measured during the study period and a conservative estimate of 750 subjects per group, the study would have 80% power to detect an increase to 20.5% in the intervention arm and 90% power to detect an increase to 21.5% in the intervention arm.

## Results

**Figure 1:**
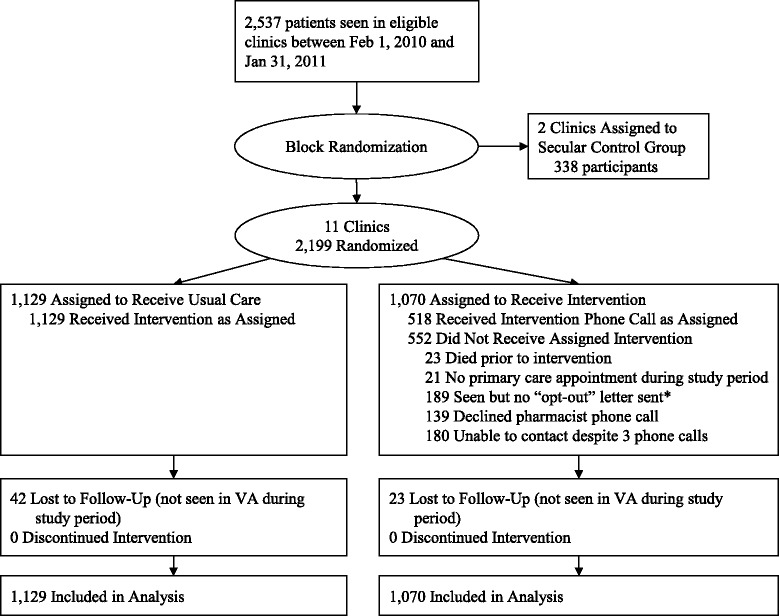
CONSORT flow diagram: Progress of patients Through out the trial. Legend. *Participants were either seen during the first few weeks of the intervention when the pharmacists’ capacity to intervene was exceeded or were seen on an urgent basis which precluded sending the “opt-out” letter at least two weeks prior to their appointment.

There were 44,698 patients seen in eligible CBOCs between February 1, 2010 and January 31, 2011. The majority of these patients did not have CKD or their most recent eGFR was greater than 45 mL/min/1.73 m^2^; 2,199 patients met inclusion and exclusion criteria and were randomized to the usual care or intervention arms of the study (see Figure 1). Baseline characteristics of the subjects are shown in Table 1.

**Table 1:** **Baseline characteristics by study group**

	**Control (N = 1129)**	**Intervention (N = 1070)**	***P*** **value**
Age (years)	75.7 (8.2)	75.6 (8.2)	0.88
Gender (N (%) male)	1106 (98.0)	1054 (98.5)	0.34
Race (N (%) black)	56 (5.0)	62 (5.8)	0.39
Comorbidities (N (%))			
Diabetes	539 (47.7)	545 (50.9)	0.13
Hypertension	958 (84.9)	925 (86.4)	0.29
Coronary artery disease	139 (12.3)	133 (12.4)	0.9
Heart failure	73 (6.5)	64 (6.0)	0.64
Systolic blood pressure (mmHg)	130.1 (18)	131.0 (18)	0.20
Diastolic blood pressure (mmHg)	70.5 (9.9)	70.7 (9.9)	0.68
Blood pressure > 130/80 (N (%))	473 (41.9)	474 (44.3)	0.26
eGFR (mL/min/1.73 m^2^)	34.5 (7.3)	34.2 (7.7)	0.32
eGFR (N (%))			
>30 mL/min/1.73 m^2^	880 (78.2)	807 (75.8)	
20-30 mL/min/1.73 m^2^	194 (17.2)	192 (18.0)	0.21
<20 mL/min/1.73 m^2^	52 (4.6)	66 (6.2)	
Proteinuria (N (%))*	179 (15.9)	200 (18.7)	0.08
Measured in the past year (N (%))			
PTH	161 (14.3)	157 (14.7)	0.78
Phosphorus	586 (51.9)	561 (52.4)	0.81
Ualb/Cr ratio	504 (44.6)	483 (45.1)	0.81
Classes of antihypertensives (N (%))^†^			
0	37 (3.9)	41 (4.4)	
1	95 (9.9)	86 (9.3)	
2	247 (25.8)	226 (24.4)	0.78
3	284 (29.6)	295 (31.9)	
4+	295 (30.8)	277 (29.9)	
Medication Adherence	1.3 (1.4)	1.2 (1.3)	0.61
Quality of Life (phone survey)			
SF12 MCS	49.8 (10.7)	49.5 (12.3)	0.88
SF12 PCS	36.8 (11.2)	39.3 (10.9)	0.12
KDQOL Burden	76.5 (25.9)	75.5 (27.1)	0.80
KDQOL Effects	84.4 (15.0)	85.3 (15.2)	0.67

Note: Values are mean (SD) except as noted. Conversion factors for units: eGFR in mL/min/1.73 m^2^ to mL/s/1.73 m^2^, multiply by 0.0167.

*UA positive for protein or Ualb/Cr >300 mg/g.

^†^for those with hypertension.

The “opt-out” letter was sent to 838 (78.3%) subjects in the intervention arm. Reasons for not sending a letter include no eligible primary care appointment during the study period and death. Additionally, during the first few weeks of the study, the number of intervention subjects with primary care appointments exceeded the capacity of the clinical pharmacists to conduct the intervention (two pharmacists working 1 day each were able to intervene on a maximum of 25 participants per week). During these first few weeks of the study, participants with poorly controlled BP and those not receiving guideline recommended care were given priority. Therefore, 232 participants in the intervention arm were not sent an “opt-out” letter.

Of the 838 intervention participants who were sent an “opt-out” letter, 139 declined the pharmacist phone call, the pharmacists were unable to contact 180 participants despite three attempts, and the pharmacists completed the phone call portion of the intervention for 518 participants (see Figure 1). Of these 518 subjects, 165 declined further educational resources while 353 were sent the “Chronic Kidney Disease: What Does it Mean for Me?” pamphlet from NKDEP. All 1070 subjects randomized to the intervention arm were included in the primary analyses.

The primary clinical outcome, last systolic BP during the study period among participants with a baseline BP > 130/80 mmHg, did not differ between the control and intervention arms (134.4 mmHg vs. 135.1 mmHg; *P* = 0.57) (see Table 2). Results were similar after controlling for baseline BP and when analyses were restricted to those with baseline BP > 140/90 mmHg. There was also no difference between the control and intervention arms for any of the secondary clinical outcomes of BP control, ESRD, death, quality of life, or CKD burden/effects.

**Table 2:** **Clinical and process outcomes**

	**Control**	**Intervention**	**P value**
**Primary clinical outcome**			
Systolic blood pressure (mmHg) among participants with baseline BP >130/80mmHg^a^	134.4 (17.6)	135.1 (17.4)	0.57
**Primary process measure**			
PTH measured during study period	182 (16.1%)	502 (46.9%)	<0.001
**Secondary clinical outcomes**			
Blood pressure < 130/80 mmHg*	177/429 (41.2%)	185/441 (42.0%)	0.84
ESRD	20 (1.8%)	26 (2.4%)	0.28
Death	74 (6.6%)	50 (4.7%)	0.06
Quality of Life (phone survey)			
SF12 MCS	52.1 (9.6)	52.0 (10.6)	0.9
SF12 PCS	36.8 (10.3)	39.3 (9.8)	0.15
KDQOL Burden	89.4 (19.6)	89.7 (20.5)	0.93
KDQOL Effects	94.4 (14.0)	94.2 (11.9)	0.92
**Secondary process measures**			
Classes of antihypertensives*			
0	65 (13.7%)	37 (7.8%)	
1	63 (13.3%)	52 (11.0%)	
2	105 (22.2%)	128 (27.0%)	0.02
3	121 (25.6%)	135 (28.5%)	
4+	119 (25.2%)	122 (25.7%)	
Measured during study period			
Phosphorus	527 (46.7%)	680 (63.6%)	<0.001
Ualb/Cr ratio	435 (38.5%)	602 (56.3%)	<0.001
Treatments			
ACEI/ARB^†^	298/483 (61.7%)	309/481 (64.2%)	0.41
Phosphorus binder‡	19/81 (23.5%)	24/107 (22.4%)	0.87
Vitamin D^§^	218/416 (52.4%)	310/501 (61.9%)	0.004
Bicarbonate^‖^	18/137 (13%)	31/132 (24%)	0.03
Medication Adherence	6.8 (1.2)	6.7 (1.2)	0.70
Seen by nephrology during study^¶^	56/246 (22.8%)	59/258 (22.9%)	0.9

Mean (SD), N (%) as appropriate.

*Baseline BP >130/80 mmHg (N = 473 for control and 474 for intervention arms; no BP obtained during the study period for 44 control and 33 intervention participants).

^†^Proteinuria or diabetes and maximum potassium ≤ 5.5 mEq/L (N = 483 and 481).

^‡^Baseline or study phosphorus > 4.6 mEq/L (N = 81 and 107).

^§^Baseline or study PTH > 110 or baseline or study 25Vit D < 30 (N = 416 and 501).

Baseline or study bicarbonate < 21 mEq/L (N = 137 and 132).

^¶^Baseline eGFR < 30 (N = 246 and 258).

The intervention was associated with a significant improvement in the primary process outcome, measurement of PTH during the study period (16.1% in the control arm vs. 46.9% in the intervention arm; *P* <0.001). Additionally, while the intervention was not associated with reduction in BP among those poorly controlled at baseline, at the end of the study, subjects in the intervention arm were prescribed more classes of antihypertensive medications than those in the control arm (*P* = 0.02). The intervention also increased the percent of subjects with a phosphorus and urine albumin to creatinine ratio measured during the study period. Subjects in the intervention arm were more likely to be appropriately treated with vitamin D and bicarbonate but there was no difference in appropriate use of ACEI/ARBs or phosphorus binders. Finally, subjects reported excellent medication adherence, as assessed by the Morisky Medication Scale, and there was no difference between the two treatment groups (Table 2). Results were similar in secondary analyses using mixed model regression. Finally, in order to assess secular trends, patients from the two CBOCs that were block randomized to a separate control group were compared to those assigned to usual care; no differences were observed with respect to the primary clinical or process outcomes.

Satisfaction with the intervention was very positive; 92% of participants who were surveyed felt that the pharmacists provided useful information and would recommend the program to others. Representative comments from participant surveys are shown in Table 3.

**Table 3:** **Participant comments regarding the pharmacist intervention**

**Participant**	**Comment**
1	“That they still know who I am, they've taken a personal interest in me, you're not just a person walking in and out”.
2	“They're looking at me as an individual, individual attention is good, hard to find”.
3	“The pharmacist knew about my medication regimen”.
4	“The VA cared enough to call”.
5	“Liked how they explained how my blood pressure should be”.
6	“It's nice that someone takes the time to call”.
7	“They told me what I should do and shouldn't do - a good reminder”.
8	“Truthfulness; the pharmacist was very good about explaining concepts and answering questions”.
9	“I liked that she reminded me to get labs, and made me remember to fill my medications”.

## Discussion

This pragmatic randomized controlled trial demonstrates that a short-term multifaceted intervention including a limited pharmacist intervention, pharmacist-physician collaboration, a CKD registry, and patient education improved adherence to KDOQI guidelines for process measures but not clinical outcomes. This clinical trial demonstrates the feasibility of a pharmacist based intervention combined with pharmacist-physician collaboration for patients with CKD. While the intervention was not associated with a reduction in BP, intervention subjects were on more antihypertensive medications at the end of the study; longer follow-up may have demonstrated improvement in BP control.

Consistent with previous studies that evaluated new models for delivering care to patients with CKD, our intervention was associated with improvement in process but not outcome measures [23-26]. Although, the MASTERPLAN study did demonstrate a reduction in renal events with a nurse practitioner based intervention in long-term follow up [27]. Similar to our results, a CKD checklist provided to primary care physicians improved guideline adherence but not blood pressure control in a non-randomized quality improvement study [26]. There are important differences between our study and prior trials. First, while our intervention focused on CKD specific processes and outcomes, previous interventions targeted both CKD and cardiovascular risk factors [23-25]. Second, the intensity of the current intervention which involved only an initial phone call with laboratory follow-up, was lower than previous designs [23,24]. Prior interventions were also clinic based as opposed to the current study which was telephone based. Finally, the one-year duration of the present study was shorter than the previous trials. Our study demonstrates that a less-intense, phone-based intervention may be sufficient to improve processes of care. Improving processes of care may not necessarily translate to improvement in patient outcomes; longer term prospective studies are needed to determine whether similar interventions reduce adverse outcomes such as progression of CKD and cardiovascular events.

This is the first trial to evaluate a pharmacist-physician collaborative intervention in CKD patients in the primary care setting. A number of studies have demonstrated the benefit of pharmacists’ interventions in the setting of kidney transplant and hemodialysis [28]. Previous trials with more intense interventions have also demonstrated that pharmacist-physician collaborations are effective at lowering BP [29-31]. In previous studies, pharmacists were located within the primary care clinics. This is the first study to evaluate the effect of a phone-based pharmacist intervention. While the current pharmacist-physician collaborative intervention did not lower BP, participants in the intervention group with poorly controlled baseline BP were on more antihypertensive medications at the end of the study than those in the control group. Primary care physicians may have increased antihypertensive medications at participants’ last clinic visit during the study period and an improvement may have been observed in the intervention arm had the study period been extended to capture follow up BPs. The reductions in BP observed in previous studies may be due in part to the fact that pharmacists were allowed to actively manage BP based on treatment algorithms as opposed to simply making recommendations in the EMR in the current study [29,31].

There are limitations to the current study. First, pharmacists were unable to intervene on all participants due to the fact that some participants did not have an eligible primary care visit during the study period and that each pharmacist, working one day per week, was able to intervene on only 12 subjects per week. Our intervention rate was similar to that observed in the pragmatic Adherence and Intensification of Medications trial which also demonstrated an increase in medication changes with no long term improvement in BP control [32]. Measurement of BP for our pragmatic trial was conducted in the clinic with no standardization of measurements [33]. Medication adherence was high at baseline and therefore difficult to improve. On the other hand, adherence to a low salt diet and home BP monitoring was low; combining the pharmacist intervention with a behavioral intervention may improve patient engagement and self-care behaviors [34]. The clinical pharmacist did not make dose titrations for antihypertensive medications, instead recommendations were made in the medical record and the primary provider was alerted. If providers develop “alert fatigue” or fail to address the recommendations, the benefits of the limited intervention on BP control may not be realized. Lastly, the intervention focused on CKD specific care. While PTH adherence is likely a good measure of CKD guideline adherence, the impact on clinical outcomes is unknown. Measurement of PTH during the study period was associated with increased number of antihypertensive medications among patients with poorly controlled BP at baseline; measurement of phosphorus and urine albumin to creatinine ratio during the study period; and appropriate treatment with ACEI/ARBs, phosphorus binders, Vitamin D, and bicarbonate (data not shown). Given the large burden of cardiovascular disease in patients with CKD, a more comprehensive approach targeting both CKD and cardiovascular risk factors may be more appropriate. Finally, despite the relatively controlled VA environment, only 23% of eligible patients were seen in a nephrology clinic at the VA. This may be in part due to the short duration of follow up and some patients may have been seen by a community nephrologist.

There are a number of strengths to the present study. It is the first to demonstrate the feasibility of pharmacist-physician collaboration in the setting of CKD. The telephone based intervention was well received by patients. Because the study was determined to be minimal risk and a waiver of consent was granted, all eligible patients were randomized which enhances the generalizability of the study. The results of this pragmatic clinical trial can be used to assess resource requirements for future pharmacist-physician collaborative interventions. The study demonstrates the utility of an EMR based chronic disease registry for identifying patients for a targeted intervention in the primary care setting. Finally, in addition to the primary clinical and process outcomes, we assessed quality of life, burden of CKD, and acceptability of the intervention.

## Conclusions

In conclusion, a multifactorial intervention centered on pharmacist-physician collaboration improved processes of care but did not result in reduction in BP. This study demonstrates the feasibility of pharmacist-primary care provider collaboration in the setting of CKD.

## Additional files

Additional file 1:
**Phone script embedded within the CKD registry utilized by pharmacists.**
Additional file 2:
**Guide to lab frequency and protocol for laboratory results.**
Additional file 3:
**National Kidney Disease Education Program – Chronic Kidney Disease: What Does it Mean for Me?.**

## Abbreviations

CKD: Chronic kidney disease
BP: Blood pressure
PTH: Parathyroid hormone
EMR: Electronic medical record
CBOCs: Community Based Outpatient Clinics
VAMC: Veterans Affairs Medical Center
eGFR: Estimated glomerular filtration rate
ESRD: End-stage renal disease
KDQOL: Kidney Disease Quality of Life
SF12: Short Form-12
MCS: Mental Component Summary
PCS: Physical Component Summary
