# Supplementary material for: A pharmacist based intervention to improve the care of patients with CKD: a pragmatic, randomized, controlled trial
Source: BMC Nephrol. 2015 Apr 16;16:56. doi: 10.1186/s12882-015-0052-2 (PMC4405859; doi:10.1186/s12882-015-0052-2)
Supplement: Additional file 1: — Phone script embedded within the CKD registry utilized by pharmacists. [file 12882_2015_52_MOESM1_ESM.docx]

Appendix 1. Phone script embedded within the CKD registry utilized by pharmacists

**Arial bold text is read by pharmacist.**

Times New Roman text includes patient answers and instructions to the pharmacist.

**Hello, my name is *[Pharmacist’s name]*, I’m calling from the Cleveland VA. I’m working with the medical team to improve the care of veterans. You have an appointment coming up with your doctor in *[name of clinic]*.**

1. **Do you have a few minutes to talk about chronic kidney disease?**
   1. Yes. Continue to #2.
   2. No. **When would be a good time for me to call you back?**
2. **Has your doctor ever told you that you have kidney disease?**
   1. Yes. Continue to #3.
   2. No. Continue to #4.
3. **So, as you may know, your kidneys are not working normally. They are functioning at about *[GFR]* percent of normal.** (go to #5)
4. **Your kidneys are not working normally. They are currently functioning at about *[GFR]* percent of normal.** (go to #5)
5. Edited from NKF:
   1. **Normal kidneys remove wastes and fluid from your body. In addition, the kidneys perform other important jobs. These include:**
      1. **Regulation of your body water and other chemicals in your blood such as sodium, potassium, phosphorus and calcium**
      2. **Removing drugs and toxins introduced into your body**
      3. **Make hormones to help your body: regulate blood pressure, make red blood cells, and promote strong bones.**
   2. **Chronic kidney disease includes conditions that damage your kidneys and decrease their ability to keep you healthy by doing the jobs listed. If kidney disease gets worse, wastes can build to high levels in your blood and make you feel sick. You may develop complications like high blood pressure, anemia (low blood count), weak bones, poor nutritional health and nerve damage. Also, kidney disease increases your risk of having heart and blood vessel disease. These problems may happen slowly over a long period of time. Chronic kidney disease may be caused by diabetes, high blood pressure and other disorders. Early detection and treatment can often keep chronic kidney disease from getting worse. If it’s ok with you, I’ll send you some information about chronic kidney disease.**
      1. OK – send patient NKDEP CKD information. (go to #6)
      2. Declines. (go to #6)
6. Does the patient have high blood pressure?
   1. No. Go to #12.
   2. Yes.
      1. Is the last systolic blood pressure < 130?
         1. Yes. Go to #12.
         2. No. Go to #7.
7. **One of the most important things you can do to help your kidneys is to maintain as normal a blood pressure as possible.**
8. **Do you follow a low salt diet?**
   1. Yes. Go to #9.
   2. No. **A low salt diet is important for treating high blood pressure. I can have a nutritionist call you to discuss how to reduce the salt in your diet. Would that be OK?**
      1. Yes/OK. Order a nutrition consult.
      2. No. Go to #9.
9. **Do you check your blood pressure at home?**
   1. Yes. **What are your most recent readings?** (Focus on the SBP, the “top” number.)
      1. Most recent SBP < 130. Go to #12.
      2. Most recent SBP > 130. Go to #10.
   2. No. **Would it be OK if I ordered a BP cuff for you?**
      1. Yes. Order a BP cuff. Go to #10.
      2. No. Go to #10.
10. **Do you have any dizziness, lightheadedness, or loss of consciousness?**
    1. Yes. Go to #12.
    2. No. Go to #11.
11. **What blood pressure medications are you taking? (What medications are you taking for your hypertension?)**
    1. If possible, increase dose of one blood pressure medication.
    2. Consider addition of new blood pressure medication.
       1. If not on calcium channel blocker, consider amlodipine 5mg daily.
       2. Other options…
    3. **Do you ever have any problems taking your medications?**
       1. Yes. **What are some of the things that make it difficult to take your medications?**
          1. After discussing barriers to medication adherence, go to 11d.
       2. No. Go to 11d.
    4. **Keeping your blood pressure controlled is very important to your health. When you go to see your doctor, ask if your blood pressure is well controlled and ask if there is anything you can do to keep your blood pressure at goal.**
12. **It is important to monitor patients with kidney disease for complications.**
13. IS THE PATIENT DUE FOR LABS?
    1. No. Go to #14. **Your doctor has been checking your labs very closely.**
    2. Yes. **After your appointment with your doctor, please go to the lab so that we can have blood drawn to check to see if you have any complications from your kidney disease.**
14. IS THE PATIENT’S GFR < 30?
    1. No. Go to #15.
    2. Yes. **Have you ever seen a kidney doctor (nephrologist)? (When was the last time?)**
       1. If never or not in the last year: **It is important for you to be seen by a kidney doctor. Can I arrange for someone to call you to make an appointment for you to be seen in our kidney clinic?**
          1. Yes. Make [renal division secretary] a co-signer on the CPRS note.
          2. No.
15. **Do you have any questions?**
16. **Thank you for taking the time to talk with me. Good-bye.**
