# Supplementary material for: A pharmacist based intervention to improve the care of patients with CKD: a pragmatic, randomized, controlled trial
Source: BMC Nephrol. 2015 Apr 16;16:56. doi: 10.1186/s12882-015-0052-2 (PMC4405859; doi:10.1186/s12882-015-0052-2)
Supplement: Additional file 2: — Guide to lab frequency and protocol for laboratory results. [file 12882_2015_52_MOESM2_ESM.docx]

Appendix 2. Guide to lab frequency and protocol for laboratory results

Desired lab frequency (at least this often)

| **Labs** | **eGFR >30** | **eGFR <30** |
| --- | --- | --- |
| K, HCO3, BUN, Cr, Ca, phos, albumin, PTH, Vit D, Hgb | Every 12 months | Every 6 months |
| If Hgb < 12mg/dL, check TIBC% and ferritin | Every 12 months | Every 6 months |
| Ualb/Cr (mircoalbumin) | Every 12 months | Every 12 months |

Response to laboratory results

1. Bicarbonate – less than 21 – start NaHCO_3_ 650mg PO BID
2. Phosphorus – greater than 4.6
   1. Nutrition consult for low phosphorus diet
   2. Corrected calcium < 9.5
      1. Start calcium acetate 667mg PO TID with meals
   3. Corrected calcium ≥ 9.5
      1. Start lanthanum 500mg PO TID with meals or sevelamer 800mg PO TID with meals
3. Corrected calcium – greater than 9.5
   1. Stop or reduce dose of calcium based phosphorus binders
   2. Hold vitamin D
4. PTH – greater than 110
   1. Corrected calcium < 9.5
      1. eGFR 30-45
         1. Start ergocalciferol 50,000 units PO q4wks x6 doses
            1. Recommend repeat Ca/Phos/PTH in 6 months
      2. eGFR < 30
         1. Start calcitriol 0.25µg PO daily (90 day supply, no refills)
            1. Recommend repeat Ca/Phos/PTH in 3 months
5. 25 Vit D – less than 30
   1. Corrected calcium < 9.5
      1. Start ergocalciferol 50,000 units PO q4wks x6 doses
         1. Recommend repeat Ca/Phos/Vit D/PTH in 6 months (3 months for eGFR < 30)
6. Potassium
   1. Greater than 5.9
      1. Call patient to obtain repeat labs emergently (ED if necessary)
   2. Greater than 5.5
      1. Nutrition consult for low potassium diet
      2. Consider recommending:
         1. Lowering dose/stopping ACEI, ARB, spironolactone, eplerenone
         2. Increasing dose of furosemide, hydrochlorthiazide
         3. NaHCO_3_ 650mg PO BID if bicarbonate less than 22
      3. Recheck potassium in 1-2 weeks
7. Creatinine
   1. Increased more than 0.5 from previous value
      1. Recheck lab within 7-10 days
         1. Repeat also increased more than 0.5 from baseline
            1. Notify [nephrologist/principal investigator]
8. Hemoglobin < 10 – consult renal anemia clinic
9. Urine albumin/creatinine ratio >30mg/g and potassium < 4.8
   1. If systolic BP > 130, recommend addition of ACEI/ARB
   2. If systolic BP < 130, recommend switching antihypertensives to include ACEI/ARB
   3. Recommend recheck of BP and basic metabolic panel within 4 weeks of adding ACEI/ARB
