# Supplementary material for: A pharmacist based intervention to improve the care of patients with CKD: a pragmatic, randomized, controlled trial
Source: BMC Nephrol. 2015 Apr 16;16:56. doi: 10.1186/s12882-015-0052-2 (PMC4405859; doi:10.1186/s12882-015-0052-2)
Supplement: Additional file 3: — National Kidney Disease Education Program – Chronic Kidney Disease: What Does it Mean for Me?. [file 12882_2015_52_MOESM3_ESM.pdf]

Chronic Kidney Disease

What Does it Mean for Me?

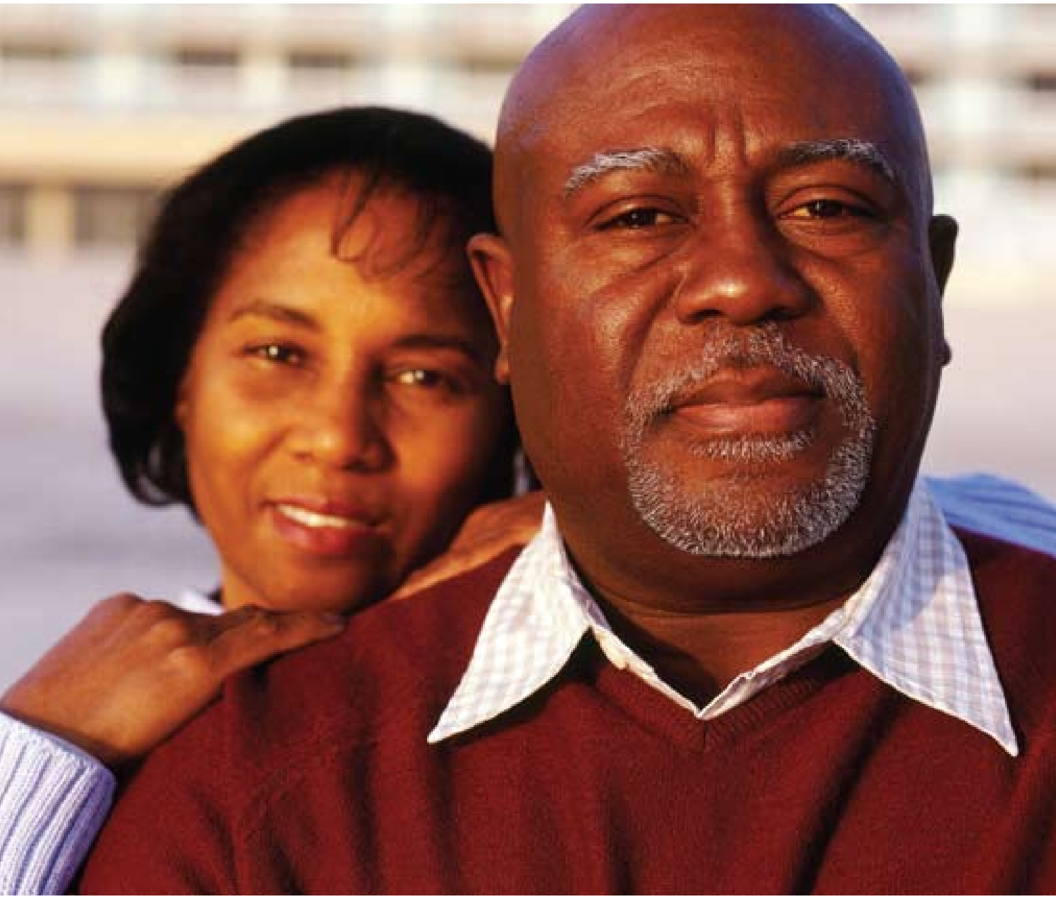

NKDEP
National Kidney Disease Education Program

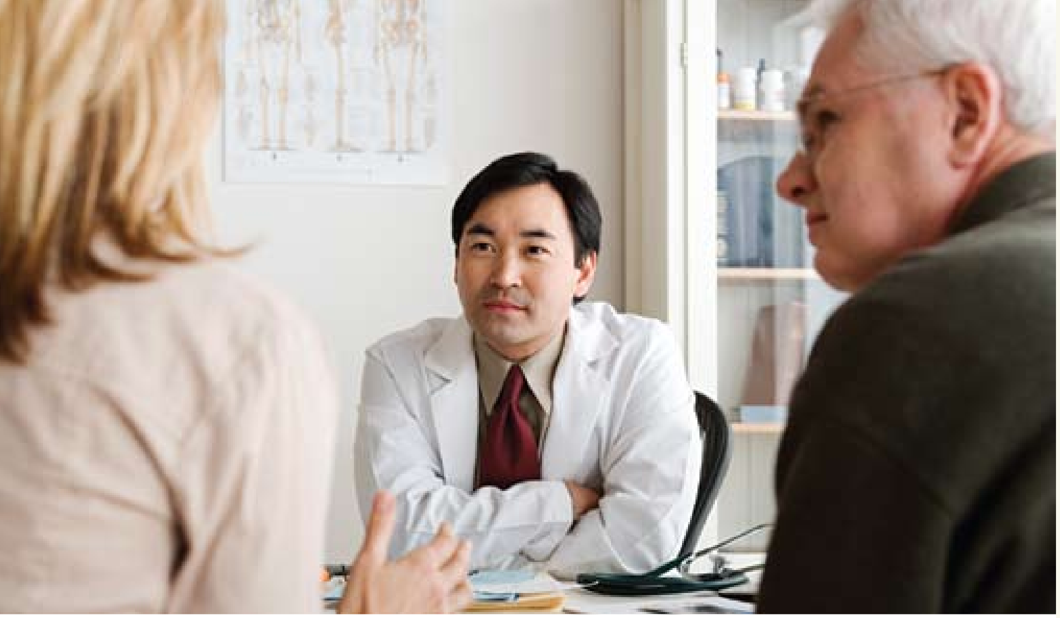

Chronic Kidney Disease: The Basics

You've been told that you have chronic kidney disease (CKD). What does that mean? And what does it mean for your health and your life? This booklet will help answer some of the questions you might have.

You have two kidneys, each about the size of your fist. Their main job is to filter wastes and excess water out of your blood to make urine. They also keep the body's chemical balance, help control blood pressure, and make hormones.

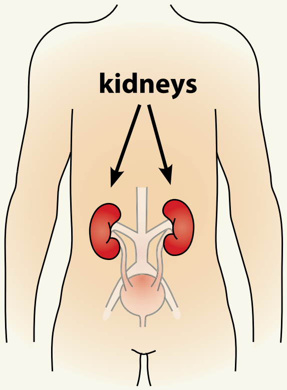

CKD means that your kidneys are damaged and can't filter blood like they should. This damage can cause wastes to build up in your body. It can also cause other problems that can harm your health.

CKD is often a "progressive" disease, which means it can get worse over time. CKD may lead to kidney failure. The only treatment options for kidney failure are dialysis or a kidney transplant.

You can take steps to keep your kidneys healthier longer:

- Choose foods with less salt (sodium).
- Keep your blood pressure below 130/80.
- Keep your blood glucose in the target range, if you have diabetes.

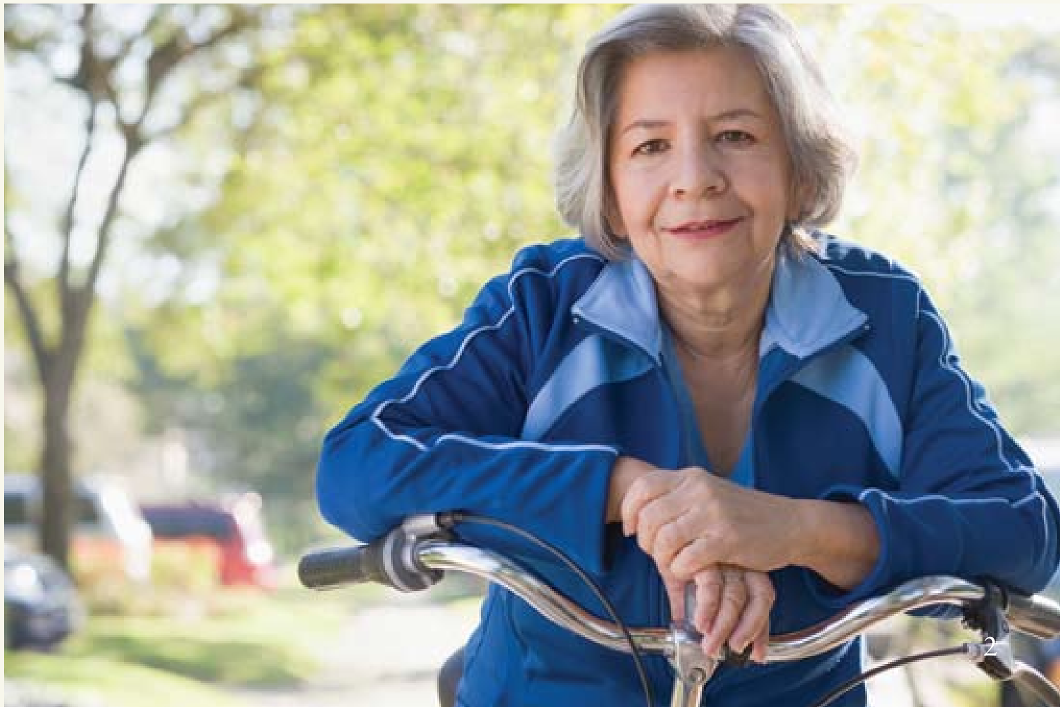

CKD and My Health

How does my health care provider know I have CKD?

Chances are, you feel normal and were surprised to hear that you have CKD. It is called a "silent" disease, because many people don't have any symptoms until their kidneys are about to fail. The only way to know is to get your kidneys checked with blood and urine tests.

1. **A blood test checks your GFR,** which tells how well your kidneys are filtering. GFR stands for glomerular filtration rate.
2. **A urine test checks for albumin.** Albumin is a protein that can pass into the urine when the kidneys are damaged. See picture below.

Inside a *healthy* kidney **Inside a *damaged* kidney**

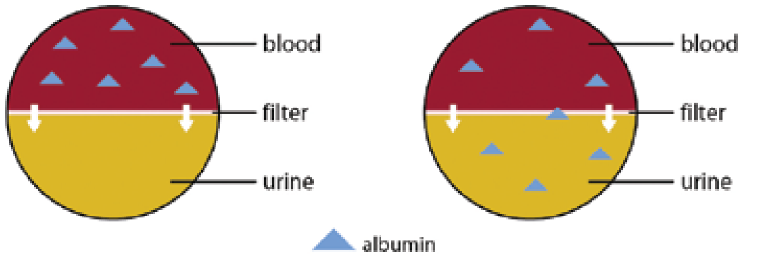

These two tests are used to monitor CKD and make sure that treatment is working. See pages 9 and 10 to learn more about these tests and track your results.

What causes CKD?

Diabetes and high blood pressure are the most common causes of CKD. There are other causes, too.

Your provider will look at your health history and may do other tests. You need to know **why** you have CKD, so your treatment can also address the cause of the CKD.

What medicines are used to treat CKD?

People with CKD often take medicines to lower blood pressure, control blood glucose, and lower blood cholesterol. Two types of blood pressure medicines—ACE inhibitors and ARBs—may slow CKD and delay kidney failure, even in people who don't have high blood pressure. Many people need to take two or more medicines for their blood pressure. They also may need to take a diuretic (water pill). The goal is to keep your blood pressure below 130/80.

Do I need to change my medicines?

Some medicines are not safe for people with CKD. Other medicines need to be taken in smaller doses. Tell your provider about all the medicines you take, including over-the-counter medicines (those you get without a prescription), vitamins, and supplements.

Can CKD affect my health in other ways?

People with CKD often have high blood pressure. They can also develop anemia (low number of red blood cells), bone disease, malnutrition, and heart and blood vessel diseases.

What tests will help track my CKD?

The blood and urine tests used to check for CKD are also used to monitor CKD. You need to keep track of your test results to see how you're doing.

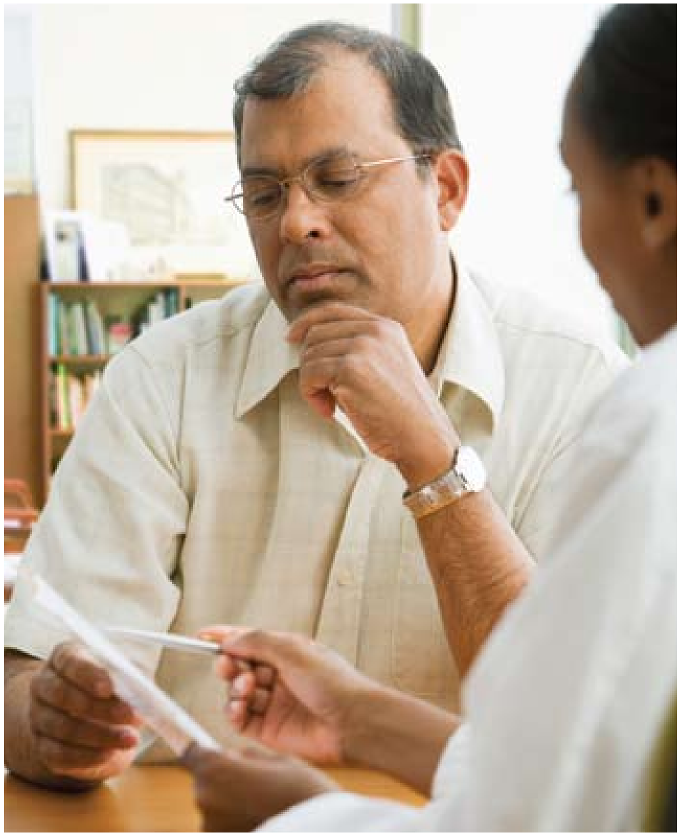

Track your blood pressure. In most cases, you should keep it below 130/80.

If you have diabetes, monitor your blood glucose and keep it in your target range. Like high blood pressure, high blood glucose can be harmful to your kidneys.

See page 9 of this booklet for more information on tracking your test results.

Will I have to go on dialysis?

Some people live with CKD for years without going on dialysis. Others progress quickly to kidney failure. You may delay dialysis if you follow your provider's advice on medicine, diet, and lifestyle changes.

If your kidneys fail, you will need dialysis or a kidney transplant. Most people with kidney failure are treated with dialysis.

Will I be able to get a kidney transplant instead of going on dialysis?

Some people with kidney failure may be able to receive a kidney transplant. The donated kidney can come from someone you don't know who has recently died, or from a living person—a relative, spouse, or friend. A kidney transplant isn't for everyone. You may have a condition that makes the transplant surgery dangerous or not likely to succeed.

CKD and My Lifestyle

People with CKD can and should continue to live their lives in a normal way: working, enjoying friends and family, and staying active. They also need to make some changes as explained here.

Do I need to change what I eat?

What you eat may help to slow down CKD and keep your body healthier. Some points to keep in mind:

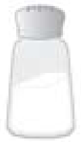	Choose and prepare foods with less salt (sodium). Use less salt at the table.
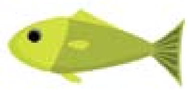	Select the right kinds and smaller amounts of protein.
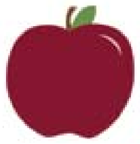	Choose foods that are healthy for your heart, like lean cuts of meat, skinless chicken, fish, fruits, vegetables, and beans.
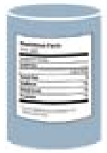	Read the Nutrition Facts Label, especially for sodium, to help you pick the right foods and drinks.

Your provider may refer you to a dietitian. Your dietitian will teach you how to choose foods that are easier on your kidneys. You will also learn about the nutrients that matter for CKD.

Do I need to change what I drink?

- Water —You don't need to drink more water unless you have kidney stones. Drink as much water as you normally do.
- Soda and other drinks — If you are told to limit phosphorus, choose light-colored soda (or pop), like lemon-lime, and homemade iced tea and lemonade. Dark-colored sodas, fruit punch, and some bottled and canned iced teas can have a lot of phosphorus.
- Juice — If you are told to limit potassium, drink apple, grape, or cranberry juice instead of orange juice.
- Alcohol — You may be able to drink small amounts of alcohol. Drinking too much can damage the liver, heart, and brain and cause serious health problems.

Is smoking cigarettes bad for my kidneys?

Take steps to quit smoking as soon as you can. Cigarette smoking can make kidney damage worse.

CKD: Tracking My Test Results

You are the most important person on your health care team. Know your test results and track them over time to see how your kidneys are doing. Bring this card to your health care visits and ask your provider to complete it.

Blood pressure — The most important thing you can do to slow down CKD is keep your blood pressure below 130/80. This can delay or prevent kidney failure.

GFR — The GFR tells you how well your kidneys are filtering blood. You can't raise your GFR. The goal is to keep your GFR from going down to prevent or delay kidney failure. See the dial picture below.

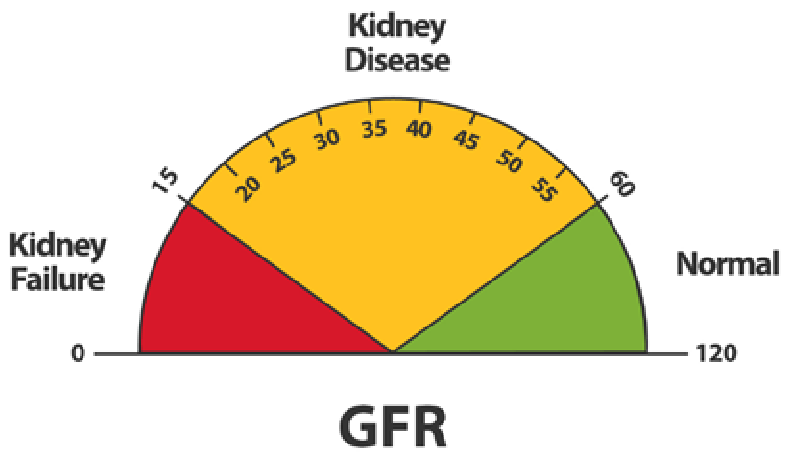

Urine albumin — Albumin is a protein in your blood that can pass into the urine when kidneys are damaged. You can't undo kidney damage, but you may be able to lower the amount of albumin in your urine with treatment. Lowering your urine albumin is good for your kidneys.

A1C — A1C test is a lab test that shows your average blood glucose level over the last 3 months. The goal is less than 7 for most people with diabetes. Lowering your A1C can help you to stay healthy. (For people with diabetes only.)

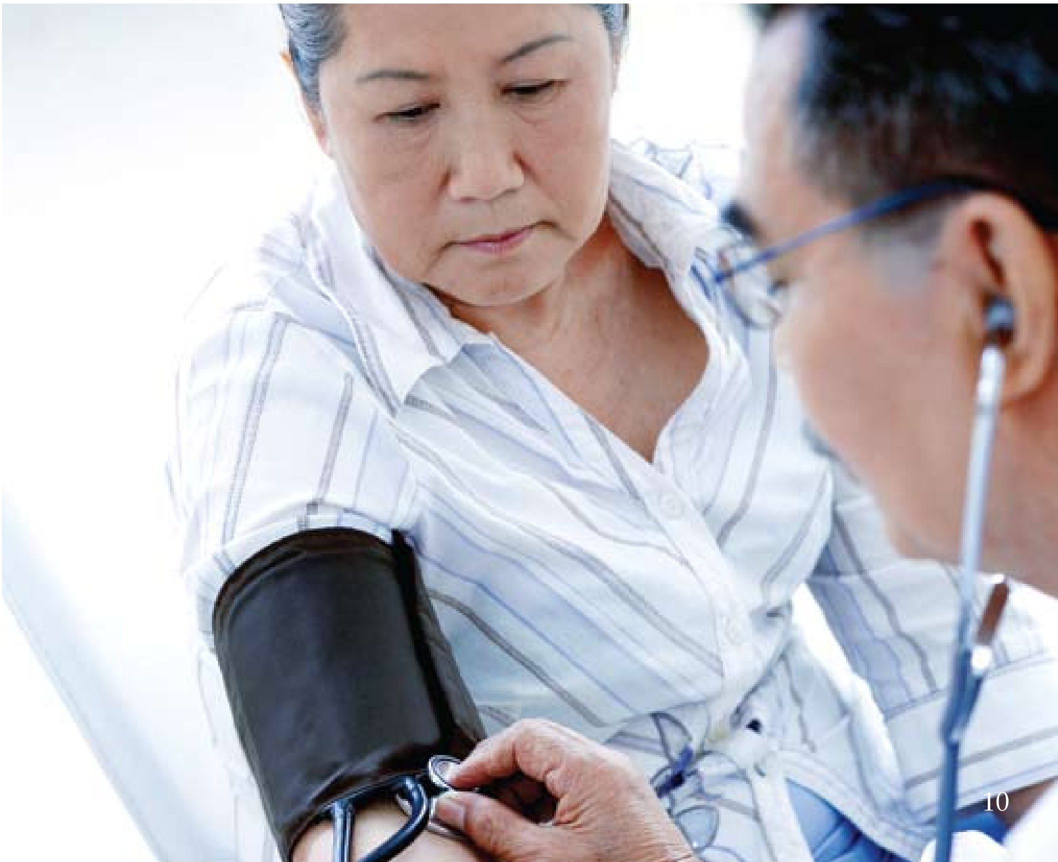

Where can I get more information?

National Kidney Disease Education Program
www.nkdep.nih.gov
1-866-4 KIDNEY (1-866-454-3639)

National Kidney and Urologic Disease Information Clearinghouse
www.kidney.niddk.nih.gov • 1-800-891-5390

American Association of Kidney Patients
www.aakp.org • 1-800-749-2257

American Kidney Fund
www.kidneyfund.org • 1-800-638-8299

National Kidney Foundation
www.kidney.org • 1-800-622-9010

Participants in clinical trials can play a more active role in their own health care, gain access to new research treatments before they are widely available, and help others by contributing to medical research. For more information, visit *www.clinicaltrials.gov*.

U.S. Department of Health and Human Services
National Institutes of Health

NIDDK NATIONAL INSTITUTE OF DIABETES AND DIGESTIVE AND KIDNEY DISEASES

NKDEP National Kidney Disease Education Program

The National Kidney Disease Education Program (NKDEP) encourages people to get tested for kidney disease and educates those with kidney disease and their health care providers about treatments that can help delay or prevent kidney failure. NKDEP is a program of the National Institutes of Health (NIH).

NIH Publication No. 10-7408 • Printed April 2010

National Kidney Disease Education Program

www.nkdep.nih.gov
1-866-454-3639 (1-866-4 KIDNEY)

Tips for People with CKD

- Choose foods with less salt
- Keep your blood pressure below 130/80
- Track your tests results
- Keep your blood glucose in the target range—if you have diabetes

My Test Results

NKDEP
National Kidney Disease Education Program

Test	Result/Date	Result/Date	Result/Date	Result/Date
**Blood pressure**<br>*Goal: Less than 130/80*				
**GFR**<br>*Goal: Keep from going down*				
**Urine albumin**<br>*Goal: The lower the better*				
**A1C (for people with diabetes)**<br>*Goal: Less than 7*				
